# Interactional practices in person‐centred care: Conversation analysis of nurse‐patient disagreement during self‐management support

**DOI:** 10.1111/hex.13236

**Published:** 2021-03-28

**Authors:** Emma Forsgren, Ida Björkman

**Affiliations:** ^1^ Institute of Health and Care Sciences Sahlgrenska Academy University of Gothenburg Gothenburg Sweden; ^2^ Centre for Person‐Centred Care (GPCC) University of Gothenburg Gothenburg Sweden

**Keywords:** conversation analysis, deontics, epistemics, nurse‐patient interaction, person‐centred care

## Abstract

**Background:**

Person‐centred care implies a change in interaction between care professionals and patients where patients are not passive recipients but co‐producers of care. The interactional practices of person‐centred care remain largely unexplored.

**Objective:**

This study focuses on the analysis of disagreements, which are described as an important part in the co‐production of knowledge in interaction.

**Design:**

A qualitative exploratory study using conversation analysis.

**Setting and participants:**

Data were collected from a nurse‐led person‐centred intervention in a hospital outpatient setting. Interactions between adult patients with irritable bowel syndrome (n = 17) and a registered nurse were audio‐recorded. COREQ guidelines were applied.

**Results:**

Disagreements were found after demonstration of the nurse's or patients’ respective professional or personal knowledge. Disagreements were also evident when deciding on strategies for self‐management. Although negotiations between opposing views of the nurse and patient were seen as important, the patient generally claimed final authority both in knowing how IBS is perceived and in the right to choose self‐management strategies. The nurse generally oriented towards patient authority, but instances of demonstration of nurse authority despite patient resistance were also found.

**Discussion and conclusions:**

This study provides information on how co‐production of knowledge and decisions occur in the context of a person‐centred care intervention. Negotiations between nurse and patient views require a flexible approach to communication, adapting interaction to each context while bearing in mind the patients having the final authority. To facilitate co‐production, the patient's role and responsibilities in interaction should be explicitly stated.

## 1 BACKGROUND

Person‐centred care (PCC), compared to usual care, has been associated with shorter hospital stays,^1^ improved self‐efficacy^2^ and quality of life.^3^ In PCC, patients serve as experts of their own health and unique living conditions, and health‐care professionals (HCP) represent generic knowledge on health, illness and care.^4^, ^5^ When power and responsibility are shared, patients become part of the health‐care team.^6^ Although PCC’s efficacy has been proved in various settings, the actual interactional practices involved need more study. This paper addresses this knowledge gap by analysing interactional data involving a registered nurse (RN) and patients with irritable bowel syndrome (IBS). The data were collected in a pilot PCC intervention for IBS that has shown potential to decrease symptom severity.^7^ Since no curative medical treatment exists and IBS is long‐term or chronic, the intervention focuses on supporting patients in their self‐management regarding diet, stress and physical activity. The intervention is based on the principles of PCC – patient narrative, partnership and documentation – as described by Ekman et al^8^ with the goal to empower the participants to actively self‐manage their IBS. Ekman et al^8^ stress the ethical base of PCC and the principles has been successfully applied in various interventions.^9^

### 1.1 Communication in person‐centred care

Health‐care services should prioritize supporting self‐management for patients with chronic or long‐term disorders and co‐production of care and health is vital to that endeavour.^10^ Although different definitions of PCC exist, all represent a shift from paternalistic to egalitarian relationships between HCP and patients – that is, a partnership in which health and care is co‐produced.^4^, ^5^, ^6^, ^11^ This partnership is created when HCP are engaged listeners, non‐judgemental and respect their patients’ personal wishes and needs.^6^, ^12^ However, HCP tend to overestimate the extent care is adapted to each person and care is often task‐oriented rather than person‐centred.^13^, ^14^

Because the scarce literature on person‐centred communication focuses mainly on dementia care (i.e. involving people with speech‐language disorders),^15^ its findings might not be transferable to other contexts and patient groups. However, more literature has focused on the related concepts of patient‐centred communication and shared decision making (SDM), which involve patients in treatment decisions. Patient‐centred communication and SDM can improve patient satisfaction^16^, ^17^ and adherence to treatment.^18^ The so‐called ‘high participation patients’ (i.e. those who express opinions) force their physicians to use a patient‐centred communication style.^19^ However, Kunneman et al^20^ report that SDM is often used in an instrumental way, ignoring humanistic aspects of interaction. Fisher et al^21^ also argue that SDM alone might not lead to patient‐centred decisions when contextual factors are ignored. SDM literature mainly ‘evaluate[s] whether or not the dancers follow the steps, not if they are dancing with each other to the music’ (Kunneman et al^20^ p.458). There might be a key difference between literature on patient‐centredness and person‐centredness reflected in the wording, where patient implies a focus on function and person a focus on living a meaningful life.^22^

### 1.2 Conversation analysis and PCC

A valuable method to investigate how people structure their interaction is Conversation Analysis (CA).^23^ CA, rooted in sociology and ethnomethodology, was developed in the early 1960s by Harvey Sacks and collaborators.^24^ CA, which is data and participant driven, relies on sequential context.^25^, ^26^ That is, the analysis takes a bottom‐up approach that examines how participants handle utterances in specific interactional contexts.

Related to co‐production in PCC, CA research explores disagreement between speakers. Disagreement has been discussed largely in relation to Pomerantz's^27^ observation that in a friendly conversation agreement is the preferred response due to the preference for interactional consensus.^27^ Therefore, disagreement could generally be seen as dispreferred, even if in some sequential positions (e.g. speaker self‐deprecation) disagreement is the preferred response. Nevertheless, recent research argues that disagreement cannot be seen as positive or negative (or preferred or dispreferred), but as a natural part of interaction when expression opposing views.^28^ Disagreement can entail intimacy and companionship between speakers.^29^, ^30^ In business meetings, disagreement can bring together opposing viewpoints.^31^ Hence, disagreement is more expected and appreciated in some contexts, which affects the form of disagreements.^28^ Pomerantz describes disagreement as a delay combined with an initial partial agreement. However, Kotthoff ^32^ shows that if disagreement escalates to heated dispute or conflict, these mitigating signals gradually disappear.

Another aspect to consider is how disagreements are enacted in interaction. Opposing views or disagreements have been discussed with respect to the epistemic and deontic status of participants.^33^, ^34^, ^35^ The epistemic status of a speaker is a condition of the person's right to knowledge within a specific domain,^36^ whereas the deontic status is a condition of the participant's right to decide how something should proceed.^37^ Superior status or authority implies both that someone claims authority and, perhaps more importantly, someone accepts that authority.^38^ Epistemic and deontic status is managed, for example, through the use of modality in interaction.^39^ In PCC, the interaction generally strives for shared status between the professional and patient as both have the right to express their respective knowledge as well as to collaborate as partners when making decisions.^33^ However, interactional equality is difficult and the professional domain is often given authority.^33^, ^35^

Using CA, this study builds on the few studies that unpack interactional practices used in PCC. The study attempts to increase the knowledge of how PCC is managed in clinical encounters. As previous research has discussed the importance of disagreement in co‐production, this study's analytical focus is on the sequential positioning and enactment of disagreement through epistemic and deontic status.

## 2 METHODS

Data were collected in a pilot project that evaluated a person‐centred intervention for patients with IBS at a hospital outpatient setting specialized in functional bowel disorders.^7^ The intervention consisted of 4 parts: individual support sessions with an RN (second author, IB) two to 4 times every second week, with additional contact by phone and/or e‐mail; health diaries; written information; and patient‐held medical records. The RN was trained in the ethics and principles for PCC but has no specific training in communication apart from what is included in a general nursing degree. The patients were referred to the clinic by their general practitioner or by self‐referral and were on a waiting list for a group education programme. Of the 105 patients on the waiting list, 36 were purposefully sampled to obtain a variation regarding age and gender. Patients were sent a letter that asked them to participate in the pilot study rather than the educational programme. Of these, 20 agreed, but two were found ineligible because of serious psychiatric disorders or insufficient language skills and one did not show up for the first session. Therefore, 17 patients were included in the study. 2 patients had mild IBS, 9 moderate and 6 severe. 2 participants were retired, 2 on full‐time sick leave, 2 unemployed, 2 students and 9 employed. All were Swedish citizens, and 2 were children of immigrants. The intervention was evaluated using interviews and questionnaires.^7^ The present analysis covered 27 interactions (audio‐recorded) between the RN and the 17 patients (4 male and 13 female; mean age 39 years).

The audio‐recordings were transcribed verbatim with sequences of interest transcribed using established CA guidelines (Appendix 1).^40^ The initial viewing of the dataset revealed an overarching pattern. First, the patients, guided by the RN’s questions, described how IBS affected their life. Second, in the following 1 or 2 encounters, the patients focused on self‐management strategies; for some patients, these discussions resulted in a collaboratively compiled health plan.

After the initial viewing, the analysis was focused on disagreement. We used Sifanous ^41^ definition of disagreement: ‘the expression of a view that differs from that expressed by another speaker’. More specifically, we focused our analysis on sequences demonstrating verbal disagreements.^27^ The analysis more specifically contained selecting target instances of disagreement. In total, 52 target instances, involving 12 participants, were identified. Each selected target instance consisted of an extract encompassing an utterance demonstrating disagreement and its preceding and following turns related to that disagreement. We conducted a turn‐by‐turn analysis of both how participants displayed and handled disagreement in sequences. Hence, the analysis included both an exploration of the situations in which disagreements appeared as well as how disagreements were negotiated in the following turns.

Collections of disagreements were then made, organized around the sequential positioning, that is the turn upon which disagreement was the response. These collections were further sorted into 2 overarching areas connected to the negotiation of disagreements in terms of demonstration of epistemic/deontic authority. The data were analysed separately by the 2 authors as well as collaboratively during monthly data sessions. To increase the reliability of the analysis, three separate data sessions were held with researchers and 2 graduate students familiar with CA.^24^

The project was approved by the regional ethics review board in Gothenburg (application no. 434‐15). All participants gave written informed consent to participate. The Consolidated criteria for reporting qualitative research (COREQ) was applied (see Data S1).^42^

## 3 RESULTS

Disagreement appeared after either the RN or patient demonstrated their knowledge on IBS symptoms or their knowledge on perceived patient resources and barriers. Both the RN and patients initiated disagreements that resulted in negotiations of epistemic authority. Disagreements were also present when developing strategies for self‐management, interactions that resulted in negotiations of deontic authority. In these situations, patient disagreements were observed after the RN provided specific strategies, whereas RN disagreement was seen after patients reported their life situation or presented possible strategies.

To enact disagreement, the RN appealed to her professional experience and knowledge and positioned herself in front of the computer. The patients appealed to their physical and emotional experience and contextual factors. Both the RN and the patients appealed to other professionals/experts to demonstrate epistemic or deontic authority. Below, representative quotations from the data are discussed.

### 3.1 Negotiations of knowledge or epistemic negotiations

#### 3.1.1 Portrayal of knowledge about IBS symptoms

Both the RN and patients initiated sequences of disagreement after demonstrating their knowledge about IBS symptoms. Patients initiated disagreement following the RN’s explanation of generic information about symptoms or models, which did not correspond with the personal experience. The RN initiated disagreements when patients were inclined to place the generic or professional explanation above their own perception.

In Extract 1, the RN and the patient (P17) discuss why the patient experiences gastric gas (Table 1).

**TABLE 1:** Extract 1

1.	RN:	no:: because when you've done these kinds measurements it's not that it's hhh necessarily that there is more gas in an IBS colon [ (.)but] it seems like you have a harder time getting it out
2.	P17:	[mh huh ]
3.	P17:	mh huh
4.	RN:	e:: for some reason so that it sort of builds up
5.	P17:	mh huh
6.	RN:	e:: and then you get this feeling that it goes [around and] like that
7.	P17:	[mh ]
8.	RN:	e:: but we don’t know why it's like that [but] they've seen that in any case
9.	P17:	[no ]
10.	P17:	Okey
11.	RN:	that with IBS you have a hard time getting rid of gas
12.	P17:	Mh
13.	RN:	Mh
14.	P17:	that£'s not how£ I experience it but it might be that (1.5) I mean ah I pass a lot =
15.	RN:	= yes yes
16.	P17:	but it might have been waiting [there for a very long time]
17.	RN:	[it has probably built ] up then =
18.	P17:	= yeah that's right =
19.	RN:	= while someone else might e: pass a little at a [time so you don't even think about it]=
20.	P17:	[yeah (1.0) that's it that's it ]
21.	RN:	= but here it builds up [until it like]=
22.	P17:	[mh mh ]
23.	RN:	= e:: a lot comes out then =
24.	P17:	= that's it =
25.	RN:	= instead of coming a little at a time [so you don't even think about it mh]
26.	P17:	[mh huh (.) mh mh ]

In a display of epistemic status, the RN tells the patient that IBS symptoms occur because the patient has problems passing gas (lines 1‐11). After listening to the RN’s description with minimal responses, potentially signalling disagreement,^27^ the patient responds by saying ‘that's not how I experience it’ (line 14). The patient uses the primary tense (i.e. tenses that express present and future time) to reflect the high truth value (or being the reality) of the statement, a strategy that claims epistemic authority over the RN.^39^ However, the patient also signals the delicacy of the situation by laughing^43^ and quickly modifies his view by stating she might be right even though this is not how he experiences IBS. The low modal operator ‘might’ downgrades the truth value of his previous statement.^39^ His simultaneously agrees and disagrees, resulting in weak disagreement.^27^ Then, the patient posits that the gas could have been collected for a long time, which the RN is quick to accept (line 18). The RN then modifies the patient's explanation to encompass both of their accounts and the patient signals agreement (lines 18‐26).

In Extract 2, the RN and the patient (P8) are talking about the patient's experiences with stress. The patient notes that several HCP have asked her about her stress (Table 2).

**TABLE 2:** Extract 2

1.	P8:	that's why I've always been like chocked >when everyone like< but how are you socially? how are you are you [stressed? and like that] and I have been like
2.	RN:	[ye:ah okey yeah ]
3.	P8:	no I [think I'm] fine you know =
4.	RN:	[no: no: ]
5.	RN:	= yeah yeah
6.	P8:	but then there is this inner stress perhaps for everything around
7.	RN:	(0.5) ye:ah e:: (.) or or it might not be tha‐ like [that's: ] =
8.	P8:	[no it varies]
9.	RN:	= because that's: that's also hm :£(almost) a problem£with the ibs group [that it] looks so very different
10.	P8:	[mh ]
11.	P8:	Mh
12.	RN:	because some have like a lot of e:: problems with [depression and] anxiety and so on =
13.	P8:	[ye:ah ]
14.	RN:	= and then that is seen in the stomach then there are those .hhh who don't have such [problems at all but anyway h‐]
15.	P8:	[yeah no I really wouldn't ] say that I'm depressed =
16.	P8:	= like [(.) nothing like that]
17.	RN:	[no:: no no ] no (0.5) but then it might be that it's more the diet [which is] e: the thing with your problems or that
18.	P8:	[m:h ]
19.	RN:	it's something connected to motor function or something we don't know
20.	P8:	no:
21	RN:	and which we can't e:: examine with the methods we have today (.) e :: m

In line 1, the patient states that she has felt shocked when HCP asked if she experiences stress, as this proposition does not align with her personal experience (or epistemic domain). The patient orients partly towards this professional view (lines 3‐6) when she suggests that ‘perhaps’ she has some inner stress. However, the use of the low modal adjunct ‘perhaps’ simultaneously downgrades the truth value of this professional account. In the following turn, the RN expresses disagreement, which, as in Extract 1, is vague.^27^ At first, the RN says ‘yes’, but she then states that ‘or it might not be’, revealing that the RN wants the patient to trust her own personal knowledge (i.e. physical and emotional experience). The RN goes on to clarify that the patient's symptoms could be connected to something else such as diet or gastrointestinal motility (lines 9‐21). The patient signals continuous agreement in the following turns, accepting the epistemic status that she knows whether she is stressed.

#### 3.1.2 Portrayal of knowledge about patient resources and barriers

Disagreement from both RN and patients was also revealed after the portrayal of patient resources and barriers. Resources and barriers refer to factors within or outside the person that either facilitate or hinder the management of IBS. Resources include strong motivation or a helpful family, and barriers include not having the strength to make changes and lacking support from others.

Extract 3 (Table 3) illustrates an interaction around a perceived barrier of a female patient (P13). Just before the extract, the conversation dealt with the patient's choice to live by herself in a secluded house because she experiences stress when in the city.

**TABLE 3:** Extract 3

1.	P13:	then I have to realise that yeah:: .hhh (1.0) I'm so:ft [ha ha ha ]=
2.	RN:	[no but that's:]=
3.	P13:	=[£I'm weak (I'm joking)£ ha ha ]=
4.	RN:	=[ha ha ha ]=
5.	RN:	=[but I also think that] e:: sometimes when I sit here and talk to: people who have stomach aches and you get stressed out by one thing or the other that (0.6) eh aren't these completely natural reactions to some kind of unnatural lifestyle that we have
6.	P13:	=[ha ha ha ]
7.	P13:	Mh
8.	RN:	that's what I'm thinking (.) this is not a natural environment to be in (name of shopping mall) [(.)then you get ]=
9.	P13:	[(no actually it's not)]
10.	RN:	= you get exhausted on [x‐ ]
11.	P13:	[yeah] you really shouldn't be that strong
12.	RN:	no [no:: ]
13.	P13:	[that's] completely true
14.	RN:	so maybe it's perhaps completely natural not to have the energy to walk around town
15.	P13:	No

The patient states that she is ‘weak’ and follows this with laughter (lines 1‐3), a signal that she interprets her situation being somewhat atypical.^43^ Schöpf et al^44^ describe laughter as a means to deliver an emotional message without losing face. In her turn, the RN shares the laughter, signalling interactional symmetry and face saving. The RN then appeals to her professional knowledge of other patients to resist the patient's assessment of being weak (lines 5‐7). The RN proposes that lack of participation in society is not weakness and a shopping mall is not a natural context. This proposition is constructed with the low modal adjuncts ‘I *think*’ (line 5) and ‘I’m *thinking*’ (line 7): the RN constructs her turn in a way that despite her disagreement acknowledges the patient as being the one with epistemic authority (i.e. the one who ultimately knows whether she is weak). In the utterance ‘aren't these completely natural reactions?’, the RN uses the term ‘completely’ to stress that the patient's feeling are natural. The RN proposes an alternate suggestion towards the patient's feeling of stress (i.e. she suggests that the patient is not weak, but completely normal), but still invites the patient's acceptance, elaboration or resistance towards her proposition. The patient accepts this proposition (lines 8‐14).

In sum, our data reveal disagreement directly following domain‐specific knowledge being portrayed, both regarding IBS symptoms and barriers and resources. Generally, the patient's experience is valued as having higher epistemic status in this setting compared to the professional knowledge. Nevertheless, the professional domain functions as an interactional tool in negotiating explanations with the patient; in Extract 1, the tool results in an explanation that considers both domains. In addition, the RN’s view of resources and barriers seems even more important as people often have a hard time assessing their strengths and weaknesses.

### 3.2 Negotiations of strategies for self‐management or deontic negotiations

#### 3.2.1 Advice on strategies for self‐management

The data reveal that patients often disagreed with the RN’s advice about self‐management. In Extract 4, the RN and a male patient (P6) discuss the use of antidepressants as a strategy for self‐management (Table 4). The patient reveals that he has stopped taking prescribed antidepressants, but the RN again states the benefits of antidepressants.

**TABLE 4:** Extract 4

1.	RN:	and then there's this with sertralin low dose antidepressant which has a very good effect on e:: pain
2.	P6:	ºyeah that's the one I tried (so it's [it e:)º]
3.	RN:	[ye:ah ] e:: =
4.	RN:	= and [the:n ]
5.	P6:	[ºbut ye:ah ] (as well yeah)º =
6.	RN:	= but you had side effects [from it or why] did you stop?
7.	P6:	[ye::ah ]
8.	P6:	ye:ah I felt e:: nauseous and e:
9.	RN:	yeah because that's often the case in the beginning [the first two] weeks there can [be: m:] some nauseousness
10.	P6:	[mh ] [ye:ah ]
11.	P6:	yeah so I felt like e: (1.0) no I felt
12.	RN:	ye:ah
13.	P6:	that it's not worth it and:: (0.6) feel so damn bad like
14.	RN:	[no ]=
15.	P6:	=[in that] way
16.	RN:	No
17.	P6:	there's this nasty nauseousness >you know<
18.	RN:	Yeah
19.	P6:	m:: yeah
20.		(1.5)
21.	RN:	e: because it's the first couple of weeks can be a little tough because e: (.) partly this with the nauseousness (0.6) but also that e:: you can get a little elevated anxiety >you can< fee:l [a little] depressed there are some m: strange effects there in the beginning but then there: hhh if you can stand the first weeks then: e: the effect can be pretty good later
22.	P6:	[mh ]
23.	P6:	mh:
24.	RN:	and then there are very low doses so it's like it's like:: like [he‐]
25.	P6:	[ah ] =
26.	P6:	= she talked about that: (name) [she] =
27.	RN:	[ah ]
28.	P6:	= is a doc[tor ] she said that also =
29.	RN:	[yeah]
30.	P6:	= but [for ] yeah =
31.	RN:	[yeah]
32.	P6:	= with that I don't sleep at night prop[erly] either =
33.	RN:	[no: ]
34.	P6:	= she said I dont want to give you a lot of sleeping pills [then because] =
35.	RN:	[no:: ]
36.	P6:	= then you are stuck in that [swamp later]
37.	RN:	[yeah(.)yeah]
		(0.6)
38.	RN:	because these e: the sertraline it's not addictive in any way (.) but it's sort o:f
39.	P6:	Mh
40.	RN:	e:: and they are well documented have been used (1.0) quite a few years and such [so::] you can think about it (.) any way
41.	RN:	[mh ]
42.	P6:	Yeah

The patient makes it clear that he has experience taking antidepressants. Explaining how the medication makes him feel ill, the patient uses primary tenses^39^ as well as words with a strong emotional connections such as ‘feel so *damn* sick’ (line 13) and this ‘*nasty* noxiousness’ (line 17). The words *damn* and *nasty* could be seen as extreme case formulations – that is, a way to legitimize his personal experience and decision not to take antidepressants.^27^ The RN accepts the patient's epistemic authority. This acceptance is demonstrated (line 21) by the use of the low modal operator ‘can’ to acknowledge: ‘[the] first couple of weeks *can* be a little tough’. This statement signals her epistemic stance as having less status (or truth value) than the patient.^39^ Nevertheless, she uses ‘little’ several times when speaking about potential side‐effects of antidepressants (i.e. ‘*little* tough’, ‘a *little* elevated anxiety levels’ and ‘a *little* depressed’), a strategy that signals her unique epistemic domain (i.e. she believes antidepressants would be helpful despite these side‐effects). The RN describes that if one can handle feeling ill for a couple of weeks, the medicine can have a positive effect. The RN suggests a low dose of a non‐addictive antidepressants to support her argument of the potential positive aspects of taking the medicine (lines 24‐38). The RN’s proposition ‘you can think about it’ (line 40) suggests that even if her view is that the patient could benefit from medication, he has the deontic authority to decide whether to take it.^37^

#### 3.2.2 Report of life situation or choice of strategies

RN disagreement was found following patient reports of life situation or self‐management strategies.

Extract 5 (Table 5) illustrates a situation involving the RN and a female patient (P2) discussing the patient's choice to exclude some foods. Previously, the patient said that her decision to exclude some foods was informed by recommendations from a famous nutritionist she saw on TV who had also written books on the subject. Using the recommendations of the nutritionist, she conducted her own tests to determine which foods (e.g. gluten) she should avoid. The extract below begins with the RN bringing the topic back to the agenda.

**TABLE 5:** Extract 5

1.	RN:	no: e:h be‐ because our dietician here she's not that keen about excluding things =
2.	P2:	= no
3.	RN:	no (.) e::m (1.0) but e:: (0.8) if you if you see a direct connection (1.0) but most often you e y‐ don't to that [perhaps]
4.	P2:	[ºnoº ]
		(0.6)
5.	RN:	so if you could try to introduce:
6.	P2:	for example pasta and such
7.	RN:	Yeah
8.	P13:	always gives me a stomachache
9.	RN:	yeah
10.	P2:	e:: that’s why I think I brought up this with gluten as well then
11.	RN:	yeah
12.	P2:	but then all the gluten I don't know [there are like] some things that: then I noticed that I ate a lot of like bread and sandwiches and that sort of stuff and it got worse but it's maybe e like (.) yeah but it could be a bit with this with constipation
13.	RN:	[no:: ]
14.	RN:	Yeah
15.	P2:	to it also makes
16.	RN:	Yeah
17.	P2:	you think about drinking a lot and
18.	RN:	yeah
19.	P2:	ºI do try to do that now butº
20.	RN:	yeah and that you maybe don't need to choose the fibre‐rich ones
21.	P2:	no
22.	RN:	No
23.	P2:	it's a bit about being able to choose the ones
24.	RN:	Yeah
25.	P2:	also not so easy to know what you should
26.	RN:	yeah that's just it it's trial and error [so you] have to test
27.	P2:	[mh ]
28.	RN:	e:: (0.8) but e: I think that it’s:: e a good way if you like >then you can< eliminate the pasta do that [definitely]
29.	P2:	[yeah ]
30.	RN:	but perhaps not everything [then] which has gluten
31.	P2:	[no ]

The RN registers her disagreement with the patient's reliance on the famous nutritionist by referring to another professional: ‘our dietician here, she's not that keen’ (line 1). The RN goes on to say that most often people do not have a direct connection between specific foods and IBS symptoms and suggests that the patient start including foods again. This request is delivered by using the qualifier ‘could’ in ‘if you *could* try’ (line 5). By constructing her proposition this way, the RN signals a contingency – that is, the patient can either accept or reject the suggestion.^45^ Hence, this request can be seen as a way to share the deontic right with the patient.^38^ The patient replies that she feels a direct connection between her IBS and pasta – in fact, she frames her experience using a high modal adjunct ‘always’.^39^ By doing this, she claims the epistemic authority as she is the one with the personal knowledge, but quickly says that she does not know whether all gluten has this effect. The extract concludes with the RN suggesting that if she excludes pasta, she should include some gluten. This proposition is delivered using the low modal adjunct ‘perhaps’, signalling the RN’s lower deontic status.^39^ Nonetheless, the RN presents a proposition in which both accounts can be included, and therefore, the proposition can be seen as a shared deontic status. The patient complies with this proposition in her final turn (lines 30‐32).

Extract 6 (Table 6) is from the end of a long interactional sequence involving a female patient (P2). The disagreement was initiated when the RN tells the patient that her work as a teacher could exacerbate IBS. During the interaction, the RN uses her epistemic status as someone with professional knowledge to claim that the patient needs to reduce her work‐related stress. Several propositions are made such as changing jobs or eating alone. In response, the patient claims epistemic authority in her personal experience and rejects the RN’s suggestions as impossible as well as ineffective. They talk about the fact that a colleague is aware of her diagnosis, and the RN asks whether her supervisor is aware (but does not elaborate on this as a possible strategy). The patient says that the supervisor is not aware of her IBS, but just moments later she returns to this topic, which the extract below illustrates.

**TABLE 6:** Extract 6

1.	P2:	I thought about what you said about my boss
2.	RN:	Yeah
		(1.0)
3.	P2:	e: that it's perhaps smart to talk to her as well because ((clears throat)) she wants to take away our morning breaks which we have
4.	RN:	Yeah
5.	P2:	she thinks like that there is too much empty time and that it should be planning time but we don't have any more planning time to schedule really
6.	RN:	no okay
7.	P2:	but e: it's e we feel that we need this little bit of [time] to like =
8.	RN:	[yeah]
9.	P2:	= be able to sit down and have a [cup of coffee and take a pee and such]=
10.	RN:	[yeah (.) yes exactly ]
11.	P2:	= and it's maybe even more important that you mention such things so that she understands that (.) [you really need it (.)]
12.	RN:	[I think so yeah ]=
13.	RN:	= because it's not like this yeah but it’s nice to have a little [break] because that's::
14.	P2:	[no ]
15.	RN:	I think that if you are going to last in [this job] then it has to in some way (.) be adapted a little to you
16.	P2:	[mh ]
17.	P2:	Mh
18.	RN:	because e:: (.) it's not worth like getting ill over a job
19.	P2:	No
20.	RN:	em:: so I think that that e:: (.) that would be great
21.	P2:	mh
22.	RN:	Mh
23.	P2:	yeah but yeah now that you mention it I haven't even reflected over that before but e:: (.) yeah

The patient says that perhaps it would be smart to talk to her manager about her situation (lines 1‐3). This account is delivered with the low modal adjunct ‘maybe’, which invites a shared deontic status,^35^ although the patient reveals that her supervisor plans to reduce breaks even further (lines 3‐12). When the patient says she could ask her supervisor not to reduce her break time, the RN gives positive feedback regarding this strategy (lines 4‐15). Her long response suggests she agrees her work conditions should be adapted (lines 16‐21). In the last line (line 24), she states this is the first time she has considered that her job should be adapted to her situation. In this interaction, the participants find a possible strategy that considers both the professional domain (stress should be reduced) and the personal domain (work conditions are difficult to change).

Extract 7 (Table 7) illustrates a sequence involving a female patient (P15). Just prior to the extract, the RN suggests that they should summarize what has been said during the session and make a plan.

**TABLE 7:** Extract 7

1.		[(5.0) ]
	RN:	[((tapping on keyboard))]
2.	RN:	e:: (2) exercise four times a week you say
3.	P15:	m huh
4.	RN:	mh:
5.		[(4.0) ]
	RN:	[((tapping on keyboard))]
6.	RN:	mh:
		(7.0)
7.		[e: (4.0) I'll write twice (2.0)] this is the e: health plan £now£ =
		[((tapping on keyboard)) ]
8.	RN:	[ha ha ha ]
9.	P15:	[okay ha ha] but [they do but they are: e:] yoga
	RN:	[((tapping on keyboard)) ]
10.	RN:	[yeah okay]
11.	P15:	[usually ] so it's a bit eas[ie ]r: workout
12.	RN:	[yeah]
13.	P15:	it's: not that inten[sive ]
14.	RN:	[no no]
15.	RN:	no I mean you should of course [work out as much as you like I'm just thinking that if you set the goal too high]=
16.	P15:	[ha ha ha]
17.		= you can like become a [little (to:) ]
18.	P15:	[yeah that’s right]
19.	RN:	eum::
20.		[(8.0) ]
21.	RN:	[((tapping on keyboard))]

The extract begins with the RN typing while both participants are silent. The RN acknowledges the patient's account of the amount of training she is involved in each week (line 2), but she demonstrates disagreement with that account and decides that the amount of exercise written in the health plan should be something else (line 8). That is, the RN claims the deontic authority using her physical positioning in front of the computer and simultaneously writing the health plan. The patient clarifies that her exercise is yoga and uses a primary tense to invoke her epistemic and deontic status as she is the one engaged in the exercise (lines 10‐14).^39^ This linguistic move resists the RN’s deontic right to determine the amount of training. The RN first says that the patient should decide on the amount of training (line 16), establishing the patient's deontic authority. The RN then says that ‘I’m just *thinking* that if one sets the goal too high one can become a little too’ and then continues to write without finishing the sentence. The modal adjunct *thinking* is a downgrading device, further stressing the patient's deontic authority. Nevertheless, this account is somewhat dubious as the RN also demonstrates her epistemic status as someone with professional knowledge when it comes to setting goals – that is, knowing the best way to formulate goals. In addition, she asserts her authority as she continues to write the health plan without the patient's input. During the RN’s account, the patient laughs, which signals an awareness of the delicate situation.^43^ Haakana^43^ notes that patients often laugh, a mark of interactional tension, when their views challenge a professional's view. In this sequence, despite the patient's initial resistance, she eventually accepts the RN’s claim to epistemic and deontic authority. This compliance with the RN’s decision is displayed by her ‘yeah that's right’ (line 19).^37^

The data reveal disagreements regarding the RN’s proposed self‐management strategies as well as the patients’ report of life situation or choice of strategies. The patient has both the epistemic authority in knowing how a proposed strategy can work in real life as well as the deontic authority regarding goals. Negotiation begins when the nurse challenges and suggests strategies. In some instances, the 2 domains cannot meet, but in some instances negotiation leads to compromises or specific strategies that the patients had not previously considered. Nevertheless, Extract 7 reveals a situation where this general strive towards patient authority is lacking, a situation that will be addressed below.

## 4 DISCUSSION

In the context of a person‐centred self‐management intervention, disagreements are connected to RN and patient knowledge and patient strategies for self‐management. Both RN and patients generally initiated disagreements as partial agreement and disagreement, resulting in vague disagreements.^27^ In negotiation of knowledge (or epistemic status), the RN clearly demonstrated that personal knowledge trumps professional knowledge; however, in discussions of self‐management goals, the RN made more claims on deontic status, resulting in the most extreme case of the RN taking authority despite resistance as in Extract 7.

As with previous research, we found that disagreement produced a back and forth movement between participants, demonstrating their knowledge while inviting the other person's account.^28^ These negotiations produced new and specific knowledge or resulted in decisions. In this process, the RN often established a weaker epistemic stance or sharing of status and the patients claimed their authority via their personal experience. The patients not only claimed epistemic and deontic authority but also accepted the RN as an authority and invited a shared status.

However, our data also demonstrate that the RN invoked her deontic right to decide what should be written in a patient's health plan by establishing her professional experience and knowledge as well as by using her physical positioning and power over documentation. This particular extract, however, can be seen as a breach of PCC as the RN ignores the patient's resistance and avoids shared status. This extract also reflects the fact that even if the patient had been given the possibility to portray her epistemic domain, the professional can ‘stumble on the final step’ – that is, produce the final version of the health plan. In PCC, a health plan should be written by the professional and patient together and either can take initiative in the actual writing.^2^

## 5 CONCLUSIONS AND IMPLICATIONS

This study provides important information on how co‐production is constructed in the context of a nurse‐led self‐management intervention, but it does not formulate a general method or technique for PCC. PCC considers the context and the persons participating in an interaction.^22^ We encourage HCP to ‘dance with their patients’, which necessitates listening to the music and not simply following predetermined steps (i.e. a generic model for communication).^20^ ‘Listening to the music’ means there is ‘no one size fits all’ when it comes to interaction – flexibility is required. This conclusion is in line with Fisher et al (2018), who stress tailoring and flexibility while supporting patients decision making.^21^ Our study confirms that HCP need to challenge their patients’ views while acknowledging that their patients have the ultimate epistemic and deontic authority.

Although our results stress interactional flexibility, some general implications can be derived. To provide opportunity for co‐production, professionals should explicitly tell their patients that their knowledge is valued and that professional knowledge should be seen as complementary (i.e. make sure that patients are aware of their rights as well as responsibilities). In addition, neither the professional nor the patient should be afraid of disagreeing with the other's account as it is in this back and forth movement that new and specific knowledge is produced. If the patients do not disagree with anything that the professional says, questions should be asked regarding how proposed strategies could work.

## CONFLICT OF INTERESTS

The authors declare that they have no known competing financial interests or personal relationships that could have appeared to influence the work reported in this paper.

## Supporting information

Table S1‐S7

Data S1

## Data Availability

The data that support the findings of this study are available from the corresponding author upon reasonable request. The data are not publicly available due to privacy or ethical restrictions.

## 1

**TABLE A1:** Key to transcription symbols

[word] [word]	Overlapping utterances
=	No break or gap between utterances/lines in transcript
(.)	Brief interval
(1.5)	Interval with time elapsed indicated
word	Word is stressed
wo:rd wor::d	Prolonged vowel or consonant
ºwordº	Quieter than surrounding speech by the same speaker
>word<	Increased speaking rate (speeding up)
<word>	Decreased speaking rate (slowing down)
.hhh	In‐breath
hhh	Out‐breath
£word£	Smiley voice or suppressed laughter
(word)	Uncertain word
( )	Transcriber not able to hear what was said
wor‐	A dash indicates a cut‐off
(( ))	Comment/description from transcriber
