# Supplementary material for: Interactional practices in person‐centred care: Conversation analysis of nurse‐patient disagreement during self‐management support
Source: Health Expect. 2021 Mar 28;24(3):940–50. doi: 10.1111/hex.13236 (PMC8235886; doi:10.1111/hex.13236)
Supplement: Supplementary file 1 — Table S1‐S7 [file HEX-24-940-s001.docx]

Table 1. Extract 1.

|  | RN: | nä:: för när man har gjort sånna här mätningar så är det inte det att hhh nödvändigtvis att det bildas mer gas i en IBS-tarm [ (.) men] det verkar som att man har svårare att få ut de |
| --- | --- | --- |
|  |  | **no:: because when you’ve done these kinds measurements it’s not that it’s hhh necessarily that there is more gas in an IBS colon [ (.)but] it seems like you have a harder time getting it out** |
|  | P17: | [mh huh ] |
|  |  | **[mh huh ]** |
|  | P17: | mh huh |
|  |  | **mh huh** |
|  | RN: | e:: av nån anledning så att det liksom samlas upp |
|  |  | **e:: for some reason so that it sort of builds up** |
|  | P17: | mh huh |
|  |  | **mh huh** |
|  | RN: | e:: å så får man den här känslan av att det kör [runt å] sådär |
|  |  | **e:: and then you get this feeling that it goes [around and] like that** |
|  | P17: | [mh ] |
|  |  | **[mh ]** |
|  | RN: | e:: men vi vet inte varför det blir så [men] det har man sett i alla fall |
|  |  | **e:: but we don’t know why it’s like that [but] they’ve seen that in any case** |
|  | P17: | [nä ] |
|  |  | **[no ]** |
|  | P17: | okej |
|  |  | **okey** |
|  | RN: | att vid IBS så har man svårigheter med å bli av med gaser |
|  |  | **that with IBS you have a hard time getting rid of gas** |
|  | P17: | mh |
|  |  | **mh** |
|  | RN: | mh |
|  |  | **mh** |
|  | P17: | de £så upplever ju inte£ jag det men så kanske det är bara det att (1.5) asså ah jag släpper då ut väldigt mycket = |
|  |  | **that£’s not how£ I experience it but it might be that (1.5) I mean ah I pass a lot =** |
|  | RN: | = ja ja |
|  |  | **= yes yes** |
|  | P17: | men då kanske det har legat [där väldigt länge] |
|  |  | **But it might have been waiting [there for a very long time]** |
|  | RN: | [de har nog byggts] upp då = |
|  |  | **[it has probably built ] up then =** |
|  | P17: | = ja just de = |
|  |  | **= yeah that’s right =** |
|  | RN: | = ah medans en annan kanske: släpper ut lite i [taget så att man inte ens tänker på det]= |
|  |  | **= while someone else might e: pass a little at a [time so you don’t even think about it]=** |
|  | P17: | [ja (1.0) just de just de ] |
|  |  | **[yeah (1.0) that’s it that’s it ]** |
|  | RN: | = men att här byggs det ju upp [tills det liksom]= |
|  |  | **= but here it builds up [until it like]=** |
|  | P17: | [mh mh ] |
|  |  | **[mh mh ]** |
|  | RN: | = e:: kommer jättemycket då = |
|  |  | **= e:: a lot comes out then =** |
|  | P17: | = just de = |
|  |  | **= that’s it =** |
|  | RN: | = istället för att komma lite i taget [så man kanske inte ens funderar på det mh] |
|  |  | **= instead of coming a little at a time [so you don’t even think about it mh]** |
|  | P17: | [mh huh (.) mh mh ] |
|  |  | **[ mh huh (.) mh mh ]** |

Table 2. Extract 2.

|  | P8: | de e ju därför jag ju alltid blitt såhär chockad >när alla ba< men hur har du det socialt? hur har du de är du [stressig? å: sån här] å jag har ju vart såhär |
| --- | --- | --- |
|  |  | **that's why I’ve always been like chocked >when everyone like< but how are you socially? how are you are you [stressed? and like that] and I have been like** |
|  | RN: | [a:h okey ah ] |
|  |  | **[ye:ah okey yeah ]** |
|  | P8: | näe jag [tycker jag har det] jättebra liksom = |
|  |  | **no I [think I'm] fine you know =** |
|  | RN: | [nä: nä: ] |
|  |  | **[no: no: ]** |
|  | RN: | = ah ah |
|  |  | **= yeah yeah** |
|  | P8: | men sen finns den här inre stressen kanske för allt runt omkring |
|  |  | **but then there is this inner stress perhaps for everything around** |
|  | RN: | (0.5) ja: e:: (.) eller eller så gör de inte d- asså [de: ]= |
|  |  | **(0.5) ye:ah e:: (.) or or it might not be tha- like [that’s: ] =** |
|  | P8: | [nä de e olika] |
|  |  | **[no it varies]** |
|  | RN: | = för de: de e ju också hm: £(nästan) problem£ med ibs gruppen [att de] ser så väldi olika ut |
|  |  | **= because that’s: that’s also hm:** £(**almost) a problem**£ **with the ibs group [that It] looks so very different** |
|  | P8: | [mh ] |
|  |  | **[mh ]** |
|  | P8: | mh |
|  |  | **mh** |
|  | RN: | för vissa har ju jättemycke e:: problem me nedstämdhet å [ångest å så] vidare = |
|  |  | **because some have like a lot of e:: problems with [depression and] anxiety and so on =** |
|  | P8: | [a:h ] |
|  |  | **[ye:ah ]** |
|  | RN: | å då visar de sig på magen sen så finns det dom som .hhh inte alls har sånna [besvär å ändå h- ] |
|  |  | **and then that is seen in the stomach then there are those .hhh who don’t have such [problems at all but anyway h-]** |
|  | P8: | [ah nä jag skulle ju verkligen inte] säga att jag är nedstämd = |
|  |  | **[yeah no I really would’t ] say that I’m depressed =** |
|  | P8: | = asså [(.) ingenting så] |
|  |  | **= like [(.) nothing like that]** |
|  | RN: | [nä:: nä nä ] nä (0.5) men då kan de ju va så att de e mer kosten [som e] e: grejen me dina besvär eller att |
|  |  | **[no:: no no ] no (0.5) but then it might be that it’s more the diet [which is] e: the thing with your problems or that** |
|  | P8: | [m:h] |
|  |  | **[m:h ]** |
|  | RN: | de e nånting me motoriken eller nånting som vi inte vet |
|  |  | **it’s something connected to motor skills or something we don’t know** |
|  | P8: | nä: |
|  |  | **no:** |
|  | RN: | och som vi inte kan e:: undersöka me dom metoderna vi har idag (.) e::m |
|  |  | **and which we can’t e:: examine with the methods we have today (.) e :: m** |

Table 3. Extract 3.

|  | P13: | så måste jag ju inse att ja:: .hhh (1.0) jag är ve:k [ha ha ha ]= |
| --- | --- | --- |
|  |  | **then I have to realise that yeah:: .hhh (1.0) I´m so:ft [ha ha ha ]=** |
|  | RN: | [nä men de:]= |
|  |  | **[no but that’s:]=** |
|  | P13: | =[£jag är svag (ja skoja)£ ha ha]= |
|  |  | **=[£I’m weak (I’m joking)£ ha ha ]=** |
|  | RN: | =[ha ha ha ]= |
|  |  | **=[ha ha ha ]=** |
|  | RN: | =[jag men jag tänker också att] e:: ibland när jag sitter här och pratar me: personer som har ont i magen å man stressar å det e det ena å det andra att (0.6) eh e inte det här helt naturliga reaktioner på nån sorts onaturlig livsstil som vi har |
|  |  | **=[but I also think that] e:: sometimes when I sit here and talk to: people who have stomach aches and you get stressed out by one thing or the other that (0.6) eh aren’t these completely natural reactions to some kind of unnatural lifestyle that we have** |
|  |  | =[ha ha ha ] |
|  |  | **=[ha ha ha ]** |
|  | P13: | mh |
|  |  | **mh** |
|  | RN: | så tänker jag (.) de e inte så naturlig miljö å gå i (namn på galleria) till exempel [(.)då blir man ju ]= |
|  |  | **that’s what I’m thinking (.) this is not a natural environment to be in (name of shopping mall) [(.)then you get ]=** |
|  | P13: | [(nä egentligen inte)] |
|  |  | **[(no actually it’s not)]** |
|  | RN: | = man blir ju helt slut på [x-] |
|  |  | **= you get exhausted on [x- ]** |
|  | P13: | [ja] egentligen ska man inte va så stark |
|  |  | **[yeah] you really shouldn’t be that strong** |
|  | RN: | näe[nä:: ] |
|  |  | **no [no:: ]** |
|  | P13: | [de är] helt rätt |
|  |  | **[that’s] completely true** |
|  | RN: | så man kanske de kanske e helt naturligt att inte orka gå på stan |
|  |  | **so maybe it’s perhaps completely natural not to have the energy to walk around town** |
|  | P13: | nä |
|  |  | **no** |

Table 4. Extract 4.

|  | RN: | å sen så e de dehär med sertralin lågdos antideppresiva som har en väldi god effekt på e:: smärta |
| --- | --- | --- |
|  |  | **and then there’s this with sertralin low dose antidepressant which has a very good effect on e:: pain** |
|  | P6: | ºah de va den jag provat (så att [de:)º] |
|  |  | º**yeah that’s the one I tried (so its’ [it e:)º]** |
|  | RN: | [a:h ] e:: = |
|  |  | **[ye:ah ] e:: =** |
|  | RN: | = å [då: ] |
|  |  | **= and [the:n ]** |
|  | P6: | [ºmen ja: ] (också ja)º = |
|  |  | **[ºbut ye:ah ] (as well yeah)º =** |
|  | RN: | = men hade du bieffekter [på den eller varför] la du av? |
|  |  | **= but you had sideeffects [from it or why] did you stop?** |
|  | P6: | [a::h ] |
|  |  | **[ye::ah ]** |
|  | P6: | a:h jag kände mig e:: illamående å: |
|  |  | **ye:ah I felt e:: nauseous and e:** |
|  | RN: | ah för de gör man ofta i början [de två första] veckorna kan de [va m:] en del illamående |
|  |  | **yeah because that’s often the case in the beginning [the first two] weeks there can [be: m:] some nauseousness** |
|  | P6: | [mh ] [a:h ] |
|  |  | **[mh ] [ye:ah]** |
|  | P6: | mh så jag kände liksom att e: (1.0) nä kände jag |
|  |  | **yeah so i felt like e: (1.0) no I felt** |
|  | RN: | a:h |
|  |  | **ye:ah** |
|  | P6: | de e inte värt å:: (0.6) må så jävla dåligt liksom |
|  |  | **that it’s not worth it and:: (0.6) feel so damn bad like** |
|  | RN: | [nä ]= |
|  |  | **[no ]=** |
|  | P6: | =[på de] viset |
|  |  | **=[in that] way** |
|  | RN: | nä |
|  |  | **no** |
|  | P6: | de blir sån här otäck illamående >asså< |
|  |  | **there’s this nasty nauseousness >you know<** |
|  | RN: | ja |
|  |  | **yeah** |
|  | P6: | m:: ja |
|  |  | **m:: yeah** |
|  |  | (1.5) |
|  | RN: | e: för de e dom första veckorna kan vara lite tuffa för e: (.) dels de här me illamåendet (0.6) men också att e:: man kan få lite förhöjda ångestnivåer >man kan känna< si:g [lite] nedstämd det blir nån m: konsti effekt där i början men sen så: hhh om man står ut där de första veckerna så: e: kan man få en god effekt sen |
|  |  | **e: because it’s the first couple of weeks can be a little tough because e: (.) partly this with the nauseousness (0.6) but also that e:: you can get a little elevated anxiety >you can< fee:l [a little] depressed there are some m: strange effects there in the beginning but then there: hhh if you can stand the first weeks then: e: the effect can be pretty good later** |
|  | P6: | [mh ] |
|  |  | **[mh ]** |
|  | P6: | mh: |
|  |  | **mh:** |
|  | RN: | å sen så är de ju så låga doser så de e ju de ju:: liksom [he-] |
|  |  | **and then there are very low doses so it’s like it’s like:: like [he-]** |
|  | P6: | [ah ] = |
|  |  | **[ah ]** = |
|  | P6: | = hon prata om de: (namn) [hon]= |
|  |  | **= she talked about that: (name) [she] =** |
|  | RN: | [ah ] |
|  |  | **[yeah]** |
|  | P6: | = e läka[re] hon sa också de = |
|  |  | **= is a doc[tor] she said that also =** |
|  | RN: | [ah] |
|  |  | **[yeah]** |
|  | P6: | = men [för] ja = |
|  |  | **= but [for] yeah =** |
|  | RN: | [ah] |
|  |  | **[yeah]** |
|  | P6: | = me att ja inte sover på nätterna ordent[ligt] heller = |
|  |  | **= with that I don’t sleep at night prop[erly] either =** |
|  | RN: | [nä:] |
|  |  | **[no:]** |
|  | P6: | = sa hon ja vill inte ge dej en massa sömntabletter [då för] = |
|  |  | **= she said I don’t want to give you a lot of sleeping pills [then because] =** |
|  | RN: | [nä:: ] |
|  |  | **[no:: ]** |
|  | P6: | = då e man ju inne i de [träsket sen] |
|  |  | **= then you are stuck in that [swamp later]** |
|  | RN: | [ah(.)ah ] |
|  |  | **[yeah(.)yeah]** |
|  |  | (0.6) |
|  | RN: | för dom här e: sertralinet e ju inte beroendeframkallande på nåt sätt (.) utan de e ju: |
|  |  | **because these e: the sertraline it’s not addictive in any way (.) but it’s sort o:f** |
|  | P6: | mh |
|  |  | **mh** |
|  | RN: | e:: å dom e väldokumenterade har använts (1.0) ganska många år å sådär  [så::] du kan ju fundera över de (.) i alla fall |
|  |  | **e:: and they are well documented have been used (1.0) quite a few years and such [so::] you can think about it (.) any way** |
|  | RN: | [mh ] |
|  |  | **[mh ]** |
|  | P6: | ja |
|  |  | **yeah** |

Table 5. Extract 5.

|  | RN: | nä: e:h fö- för våran dietist här hon är inte så där pigg på att man ska utesluta saker = |
| --- | --- | --- |
|  |  | **no: e:h be- because our dietician here she’s not that keen about excluding things =** |
|  | P2: | = nä |
|  |  | **= no** |
|  | RN: | nä (.) e::m →(1.0) men e:: (0.8) om man om man upplever en direkt koppling (1.0) men oftast så e j- gör man ju inte det [kanske] |
|  |  | **no (.) e::m →(1.0) but e:: (0.8) if you if you see a direct connection (1.0) but most often you e y- don’t to that [perhaps]** |
|  | P2: | [ºnäº ] |
|  |  | **[ºnoº ]** |
|  |  | **(0.6)** |
|  | RN: | så om du kan försöka introducera: |
|  |  | **So if you could try to introduce:** |
|  | P2: | typ pasta och sånt |
|  |  | **for example pasta and such** |
|  | RN: | ah |
|  |  | **yeah** |
|  | P13: | får jag allti ont i magen av |
|  |  | **always gives me a stomach ache** |
|  | RN: | ah |
|  |  | **yeah** |
|  | P2: | e:: de är därför jag tror att jag kom in på det här lite med gluten också då |
|  |  | **e:: that’s why I think I brought up this with gluten as well then** |
|  | RN: | ah |
|  |  | **yeah** |
|  | P2: | men sen så all gluten vet jag ju inte [de är ju lik]som vissa grejer som: sen så märkte jag jag åt väldigt mycket typ bröd å mackor och sånt och det blev ju också värre men det kanske e typ (.) ja men det kanske är lite med det här med förstoppnings |
|  |  | **But then all the gluten I don’t know [there are like] some things that: then I noticed that I ate a lot of like bread and sandwiches and that sort of stuff and it got worse but it’s maybe e like (.) yeah but it could be a bit with this with constipation** |
|  | RN: | [nä:: ] |
|  |  | **[no:: ]** |
|  | RN: | ah |
|  |  | **yeah** |
|  | P2: | å göra också |
|  |  | **to it also makes** |
|  | RN: | ah |
|  |  | **yeah** |
|  | P2: | man får tänka på å dricka mycket å |
|  |  | **you think about drinking a lot and** |
|  | RN: | ah |
|  |  | **yeah** |
|  | P2: | ºdet försöker jag ju göra nu i och för sig menº |
|  |  | **ºI do try to do that now butº** |
|  | RN: | ja å att du kanske inte ta behöver ta det mest fiberrika |
|  |  | **yeah and that you maybe don’t need to choose the fibre-rich ones** |
|  | P2: | nä |
|  |  | **no** |
|  | RN: | nä |
|  |  | **no** |
|  | P2: | det är väl lite att kunna välja ut där då |
|  |  | **it’s a bit about being able to choose the ones** |
|  | RN: | ah |
|  |  | **yeah** |
|  | P2: | också lite krångligt att veta vad man ska |
|  |  | **also not so easy to know what you should** |
|  | RN: | ja det är ju det de e ju trial and error [så att man] måste testa |
|  |  | **yeah that’s just it it’s trial and error [so you] have to test** |
|  | P2: | [mh ] |
|  |  | **[mh ]** |
|  | RN: | e:: (0.8) men e: jag tror att de:: e ett bra sätt om man liksom >då kan du ju< utesluta pastan gör de [absolut] » |
|  |  | **e:: (0.8) but e: I think that it’s:: e a good way if you like >then you can< eliminate the pasta do that [definitely] »** |
|  | P2: | [ah ] |
|  |  | **[yeah ]** |
|  | RN: | men kanske inte allting [då] som har gluten |
|  |  | **but perhaps not everything [then] which has gluten** |
|  | P2: | [no] |
|  |  | **[no ]** |

Table 6. Extract 6.

|  | | P2: | | jag tänkte på det du sa med chefen | |
| --- | --- | --- | --- | --- | --- |
|  | |  | | **I thought about what you said about my boss** | |
|  | | RN: | | ah | |
|  | |  | | **yeah** | |
|  | |  | | **(1.0)** | |
|  | | P2: | | e: att det kanske egentligen e käckt att prata med henne också för att ((harklar sig)) hon vill ta bort våra förmiddagsraster som vi har då | |
|  | |  | | **e: that it’s perhaps smart to talk to her as well because ((clears throat)) she wants to take away our morning breaks which we have** | |
|  | | RN: | | ah | |
|  | |  | | **yeah** | |
|  | | P2: | | hon tycker liksom att det är för mycket hål utan då ska det ligga som planeringstid men vi har ingen mer planeringstid att lägga ut egentligen | |
|  | |  | | **she thinks like that there is too much empty time and that it should be planning time but we don’t have any more planning time to schedule really** | |
|  | | RN: | | nä okej | |
|  | |  | | **no okay** | |
|  | | P2: | | men: då e vi känner att vi behöver den här lilla [stunden] liksom att = | |
|  | |  | | **but e: it’s e we feel that we need this little bit of [time] to like =** | |
|  | | RN: | | [ah ] | |
|  | |  | | **[yeah]** | |
|  | | P2: | | = kunna sätta sig ned och ta en [kopp kaffe å kissa och sådär]= | |
|  | |  | | **= be able to sit down and have a [cup of coffee and take a pee and such]=** | |
|  | | RN: | | [ah (.) ja precis ] | |
|  | |  | | **[yeah (.) yes exactly ]** | |
|  | | P2: | | = å de kanske är ännu viktigare att man nämner då sånt här för att hon ska förstå att (.) [man verkligen behöver det ( )] | |
|  | |  | | **= and it’s maybe even more important that you mention such things so that she understands that (.) [you really need it ( )]** | |
|  | | RN: | | [jag tror det ah ]= | |
|  | |  | | **[I think so yeah ]=** | |
|  | | RN: | | = för då är det inte såhär ja men det är gött att ha lite [rast] för de:: | |
|  | |  | | **= because it’s not like this yeah but it’s nice to have a little [break] because that’s::** | |
|  | | P2: | | [nä ] | |
|  | |  | | **[no ]** | |
|  | | RN: | | jag tror att om du ska hålla i det [här jobbet] så måste det på nåt sätt (.) anpassas lite efter dig | |
|  | |  | | **I think that if you are going to last in [this job] then it has to in some way (.) be adapted a little to you** | |
|  | | P2: | | [mh ] | |
|  | |  | | **[mh ]** | |
|  | | P2: | | mh | |
|  | |  | | **mh** | |
|  | | RN: | | för att e:: (.) det e ju inte värt att liksom gå och bli sjuk över ett jobb | |
|  | |  | | **because e:: (.) it’s not worth like getting ill over a job** | |
|  | | P2: | | nä | |
|  | |  | | **no** | |
|  | | RN: | | em:: så att jag tror att de:: (.) de skulle vara jättebra | |
|  | |  | | **em:: so I think that that e:: (.) that would be great** | |
|  | | P2: | | mh | |
|  | |  | | **mh** | |
|  | | RN: | | mh | |
|  | |  | | **mh** | |
|  | | P2: | | ja men ah nu när du säger det jag har inte ens reflekterat över det innan men e:: (.) ja | |
|  | |  | | **yeah but yeah now that you mention it I haven’t even reflected over that before but e:: (.) yeah** | |
|  | |  | |  | |
|  | |  | |  | |
|  | |  | |  | |
|  | |  | |  | |
|  | |  | |  | |
|  | |  | |  | |
|  | |  | |  | |
|  | |  | |  | |
|  | |  | |  | |
|  | |  | |  | |
|  | |  | |  | |
|  | |  | |  | |
|  | |  | |  | |
|  | |  | |  | |
|  | |  | |  | |
|  | |  | |  | |
|  | |  | |  | |
|  | |  | |  | |
|  | |  | |  | |
|  | |  | |  | |
|  | |  | |  | |
|  | |  | |  | |
|  | |  | |  | |
|  | |  | |  | |
|  | |  | |  | |
|  | |  | |  | |
|  | |  | |  | |
|  | |  | |  | |
|  | |  | |  | |
|  | |  | |  | |
|  | |  | |  | |
|  | |  | |  | |
|  | |  | |  | |
|  | |  | |  | |
|  | |  | |  | |
|  | |  | |  | |
|  | |  | |  | |
|  | |  | |  | |
|  | |  | |  | |
|  | |  | |  | |
|  | |  | |  | |
|  | |  | |  | |

Table 7. Extract 7.

|  |  | **[(5.0) ]** |
| --- | --- | --- |
|  | RN: | **[((tapping on keyboard))]** |
|  | RN: | e:: (2) träna fyra gånger i veckan säger du |
|  |  | **e:: (2) exercise four times a week you say** |
|  | P15: | m huh |
|  |  | **m huh** |
|  | RN: | mh: |
|  |  | **mh:** |
|  |  | **[(4.0) ]** |
|  | RN: | **[((tapping on keyboard))]** |
|  | RN: | mh: |
|  |  | **mh:** |
|  |  | **(7.0)** |
|  |  | [e: (4.0) jag skriver två gånger(2.0)] de här e: hälsoplanen £nu då£ = |
|  |  | **[e: (4.0) I’ll write twice (2.0) ] this is the e: health plan £now£ =** |
|  |  | **[((tapping on keyboard)) ]** |
|  | RN: | [ha ha ha ] |
|  |  | **[ha ha ha ]** |
|  | P15: | [jaha ha ha] men [de gö ju men de e ju: e:] yoga |
|  |  | **[okay ha ha] but [they do but they are: e:] yoga** |
|  | RN: | **[((tapping on keyboard)) ]** |
|  | RN: | [ja okey] |
|  |  | **[yeah okay ]** |
|  | P15: | [oftast ] så det är ju lite lugn[ar]e: träning |
|  |  | **[usually ] so it’s a bit eas[ie]r: workout** |
|  | RN: | [ja] |
|  |  | **[yeah]** |
|  | P15: | de: inte så där högint[ensivt] |
|  |  | **it’s: not that inten[sive]** |
|  | RN: | [nä nä] |
|  |  | **[no no]** |
|  | RN: | nä de e klart du ska [träna hur mycke du vill men jag tänker bara att n: om man sätter målet för högt]= |
|  |  | **no I mean you should of course [work out as much as you like I’m just thinking that if you set the goal too high]=** |
|  | P15: | [ha ha ha  ] |
|  |  | **[ha ha ha**  **]** |
|  |  | = så kan man liksom bli [lite fö: ] |
|  |  | **= you can like become a [little (to:) ]** |
|  | P15: | [ah just det] |
|  |  | **[yeah that’s right]** |
|  | RN: | **eum::** |
|  |  | **[(8.0) ]** |
|  | RN: | **[((tapping on keyboard))]** |
