# Supplementary material for: Interactional practices in person‐centred care: Conversation analysis of nurse‐patient disagreement during self‐management support
Source: Health Expect. 2021 Mar 28;24(3):940–50. doi: 10.1111/hex.13236 (PMC8235886; doi:10.1111/hex.13236)
Supplement: Supplementary file 2 — Data S1 [file HEX-24-940-s002.docx]

Supporting information.

**COREQ (COnsolidated criteria for REporting Qualitative research) Checklist**

| **Topic** | **Item No.** | **Guide Questions/Description** | **Reported in section:** |
| --- | --- | --- | --- |
| **Domain 1: Research team and reflexivity** | | | |
| *Personal characteristics* | | | |
| Interviewer/facilitator | 1 | Which author/s conducted the interview or focus group? | Methods |
| Credentials | 2 | What were the researcher’s credentials? E.g. PhD, MD | Title page |
| Occupation | 3 | What was their occupation at the time of the study? | IB was a RN and doctoral student at the time of data collection |
| Gender | 4 | Was the researcher male or female? | Methods |
| Experience and training | 5 | What experience or training did the researcher have? | Title page |
| *Relationship with participants* | | | |
| Relationship established | 6 | Was a relationship established prior to study commencement? | The RN had no prior relationship to the participants. |
| Participant knowledge of the interviewer | 7 | What did the participants know about the researcher? e.g. personal goals, reasons for doing the research | RN was introduced as a research nurse with specific interest and knowledge of IBS. |
| Interviewer characteristics | 8 | What characteristics were reported about the inter viewer/facilitator? e.g. Bias, assumptions, reasons and interests in the research topic | The RN was trained in the ethics and principles for person-centred care but had no specific training in communication apart from what is included in a general nursing degree. |
| **Domain 2: Study design** | | | |
| *Theoretical framework* | | | |
| Methodological orientation and Theory | 9 | What methodological orientation was stated to underpin the study? e.g.  grounded theory, discourse analysis, ethnography, phenomenology, content analysis | Stated in the background, Conversation Analysis |
| *Participant selection* |  |  |  |
| Sampling | 10 | How were participants selected? e.g. purposive, convenience, consecutive, snowball | Stated in methods (Patients were recruited from a waiting list for a patient group education program. Of the 105 patients on the waiting list, 36 were purposively sampled. A variation regarding age and gender was strived for and recruitment were conducted at three time points to maximize the number of men). |
| Method of approach | 11 | How were participants approached? e.g. face-to-face, telephone, mail, email | Stated in methods (Approached by mail with subsequent phone call). |
| Sample size | 12 | How many participants were in the study? | Methods, n 17 |
| Non-participation | 13 | How many people refused to participate or dropped out? Reasons? | Stated in methods (Twenty patients agreed to participate but two were ineligible due to psychiatric illness and insufficient language skills. One did not show up for the first visit). |
| *Setting* |  |  |  |
| Setting of data collection | 14 | Where was the data collected? e.g. home, clinic, workplace | Stated in methods (The intervention during which data was collected consists of four parts: individual support sessions with RN two to four times every second week, with additional contact by phone and/or e-mail, health diary, written information and patient-held medical records). |
| Presence of nonparticipants | 15 | Was anyone else present besides the participants and researchers? | Methods |
| Description of sample | 16 | What are the important characteristics of the sample? e.g. demographic data, date | Stated in methods (Two had mild IBS, nine moderate IBS, and six severe IBS (measured by IBS-SSS). Two of the participants were retired, two on full-time sick leave, two unemployed, two students, and nine employed. All were of Swedish origin and two had immigrated parents). |
| *Data collection* |  |  |  |
| Interview guide | 17 | Were questions, prompts, guides provided by the authors? Was it pilot tested? | N/A |
| Repeat interviews | 18 | Were repeat inter views carried out? If yes, how many? | N/A |
| Audio/visual recording | 19 | Did the research use audio or visual recording to collect the data? | Methods |
| Field notes | 20 | Were field notes made during and/or after the interview or focus group? | N/A |
| Duration | 21 | What was the duration of the inter views or focus group? | Stated in methods (Each patient met the RN on 1-3 occasions for approximately 40-90 minutes at a hospital out-patient setting. No time limit was set for these interactions). |
| Data saturation | 22 | Was data saturation discussed? | N/A |
| Transcripts returned | 23 | Were transcripts returned to participants for comment and/or correction? | N/A |
| **Domain 3: analysis and findings** | | | |
| *Data analysis* |  |  |  |
| Number of data coders | 24 | How many data coders coded the data? | Methods |
| Description of the coding tree | 25 | Did authors provide a description of the coding tree? | N/A |
| Derivation of themes | 26 | Were themes identified in advance or derived from the data? | Background and Methods |
| Software | 27 | What software, if applicable, was used to manage the data? | No software used |
| Participant checking | 28 | Did participants provide feedback on the findings? | N/A |
| *Reporting* |  |  |  |
| Quotations presented | 29 | Were participant quotations presented to illustrate the themes/findings?  Was each quotation identified? e.g. participant number | Results |
| Data and findings consistent | 30 | Was there consistency between the data presented and the findings? | Results |
| Clarity of major themes | 31 | Were major themes clearly presented in the findings? | Results |
| Clarity of minor themes | 32 | Is there a description of diverse cases or discussion of minor themes? | N/A |

Developed from: Tong A, Sainsbury P, Craig J. Consolidated criteria for reporting qualitative research (COREQ): a 32-item checklist for interviews and focus groups. *International Journal for Quality in Health Care*. 2007. Volume 19, Number 6: pp. 349 – 357
